# NON-FUNCTIONING SPORADIC PANCREATIC NEUROENDOCRINE TUMOR IS AN INDEPENDENT RISK FACTOR FOR RECURRENCE AFTER SURGICAL TREATMENT

**DOI:** 10.1590/0102-6720202400063e1857

**Published:** 2025-01-20

**Authors:** Estela Regina Ramos Figueira, André Luis Montagnini, Jessica Okubo, Ana Gabriela Vivarelli Fernandes, Marina Alessandra Pereira, Ulysses Ribeiro, Paulo Herman, José Jukemura

**Affiliations:** 1 Universidade de São Paulo, Faculty of Medicine – São Paulo (SP), Brazil.

**Keywords:** Neuroendocrine tumors, Patient outcome assessment, Pancreas, Pancreatectomy, Tumores neuroendócrinos, Avaliação de resultados da assistência ao paciente, Pâncreas, Pancreatectomia

## Abstract

**BACKGROUND:**

Pancreatic neuroendocrine tumors (PNETs) are uncommon and heterogeneous neoplasms, often exhibiting indolent biological behavior. Their incidence is rising, largely due to the widespread use of high-resolution imaging techniques, particularly influencing the diagnosis of sporadic non-functioning tumors, which account for up to 80% of cases. While surgical resection remains the only curative option, the impact of factors such as tumor grade, size, and type on prognosis and recurrence is still unclear.

**AIMS:**

To investigate prognostic risk factors and outcomes in patients with sporadic PNETs treated surgically.

**METHODS:**

A retrospective analysis was conducted on patients with sporadic PNETs who underwent pancreatic resection. Data were collected from medical records.

**RESULTS:**

A total of 113 patients were included: 32 with non-functioning tumors (NF-PNETs), 70 with insulinomas, and 11 with other functioning tumors (OF-PNETs). Patients with insulinoma were significantly younger, had a higher BMI, lower prevalence of comorbidities and ASA scores, and underwent significantly more pancreatic enucleations compared to patients with OF-PNET and NF-PNET. The insulinoma group had more grade I tumors, smaller tumor diameter, lower TNM staging, and lower disease recurrence rates. In univariate analysis, age, tumor type, tumor size, and TNM staging were identified as potential risk factors for tumor recurrence. In multivariate analysis, only the NF-PNET type was identified as an independent prognostic factor for disease recurrence.

**CONCLUSIONS:**

NF-PNETs are an independent prognostic risk factor for disease recurrence. This finding supports the need for closer follow-up of patients with small tumors who are selected for conservative management.

## RESUMO

**RACIONAL:**

Os tumores neuroendócrinos pancreáticos (TNEP) são neoplasias raras e heterogêneas, frequentemente com comportamento biológico indolente. A incidência está aumentando, em parte devido à disseminação de técnicas de imagem de alta resolução, o que impacta o diagnóstico de tumores esporádicos não funcionantes, responsáveis por até 80% dos casos. Embora a ressecção cirúrgica seja a única opção curativa, o impacto de fatores como grau tumoral, tamanho e tipo no prognóstico e recidiva do tumor ainda é incerto.

**OBJETIVOS:**

Investigar os fatores prognósticos e desfechos de pacientes com TNEP esporádicos tratados cirurgicamente.

**MÉTODOS:**

Foram analisados, retrospectivamente, pacientes com TNEP esporádicos submetidos à ressecção pancreática. Dados foram coletados de prontuários médicos.

**RESULTADOS:**

Foram incluídos 113 pacientes: 32 com TNEP não funcionantes (TNEP-NF), 70 com insulinomas e 11 com outros tumores funcionantes (TNEP-OF). Pacientes com insulinoma eram mais jovens, com maior IMC, menor prevalência de comorbidades, menor escore ASA, e foram submetidos a maior número de enucleações pancreáticas comparados aos pacientes com TNEP-OF e TNEP-NF. O grupo de insulinoma apresentou mais tumores grau I, menor diâmetro tumoral, menor estadiamento TNM e menor recidiva da doença. Na análise univariada, idade, tipo, tamanho do tumor e TNM foram fatores de risco para recidiva. Na análise multivariada, apenas o tipo TNEP-NF foi identificado como fator prognóstico independente para recidiva.

**CONCLUSÕES:**

Tumores não funcionantes TNEP apresentam fator de risco prognóstico independente para recidiva da doença. Este achado endossa a necessidade de observação com seguimento mais rigoroso de pacientes com tumores pequenos selecionados para tratamento conservador.

## INTRODUCTION

Neuroendocrine tumors are uncommon, heterogeneous neoplasms of the endocrine system, exhibiting highly variable biological behavior, ranging from slow-growing benign tumors to aggressive malignant ones. The PNETs subgroup has an annual incidence of 0.7 cases per 100,000 people in Japan and 1.5 per 100,000 in the USA, according to data from the National Cancer Institute's Surveillance, Epidemiology, and End Results (SEER), which shows a significant increase in recent years^26,29^. The widespread use of high-resolution imaging techniques may have contributed to this increase, particularly in diagnosing of nonfunctioning tumors^22^.

Most PNETs are sporadic, although approximately 10% are associated with hereditary syndromes. Multiple endocrine neoplasia type 1 (MEN 1) is the most common syndrome linked to PNETs, accounting for 30-80% of cases. Other syndromes, including Von Hippel-Lindau disease (10-17%), Neurofibromatosis type 1 (10%), and Tuberous sclerosis (1%), are less frequently associated with PNETs^32^. Functioning PNETs present with hormone-related syndromes, while NF-PNETs are more common, representing up to 80% of cases^22^. The prevalence of NF-PNETs is rising, largely due to the increased detection of small tumors (<2 cm)^37^. This has led to a debate regarding the optimal management of these small NF-PNET lesions, with some advocating for a conservative, wait-and-see approach, while others recommend surgical intervention^15,18^.

Since 2000, the World Health Organization (WHO) and, more recently, the American Joint Committee on Cancer (AJCC) Tumor, Node, and Metastasis (TNM) staging system have been instrumental in assessing tumor prognosis and recurrence^5,14,34^. Currently, surgical resection is the only curative option, with a 5-year survival rate ranging from 44 to 87%^14^. However, surgical strategies based on histological type, grade, size, and location of PNETs are not fully standardized, ranging from tumor enucleation to extended pancreatic resections, and their prognostic outcomes remain largely unknown. Thus, this study aimed to investigate the risk factors associated with disease recurrence and the prognosis of resected PNETs.

## METHODS

This retrospective observational study was approved by the Institutional Ethics Committee (No. 140.478) with a waiver of informed consent. The study was conducted in compliance with the ethical standards of the Institution and the 1964 Helsinki Declaration and its later amendments. Patients included in the study were followed either at University Hospital or at the Cancer Institute of the School of Medicine of Universidade de São Paulo. A total of 159 patients with sporadic PNETs who underwent pancreatic resection with curative intent were assessed. Patients were excluded if they had perioperative distant metastasis, insufficient data in medical records, or tumors without histological confirmation.

### Variables analyzed

The variables analyzed included age, gender, body mass index (BMI), comorbidities, Karnofsky performance status (KPS), American Society of Anesthesiologists (ASA) physical status score, type of surgery, postoperative complications, tumor type, size, grade, lymph node and distant metastasis, TNM and WHO staging, hospital stay, mortality, and disease-free survival (DFS) interval.

### Diagnosis and staging

Diagnosis was based on the presence or absence of clinical syndromes in conjunction with pathological analysis. Tumor staging followed the 2022 WHO classification and the TNM staging system from AJCC, 9^th^ edition^6,31,34,41^.

### Surgical procedures

For small tumors, surgical options included enucleation, distal pancreatectomy with spleen preservation, or central pancreatectomy for less favorable tumor locations. Patients with suspected malignancies underwent pancreatic resections with locoregional lymphadenectomy.

### Postoperative complications and follow-up

Postoperative complications were graded according to the Clavien-Dindo classification^10^. The presence of postoperative pancreatic fistula (POPF) was diagnosed according to the International Study Group on Pancreatic Fistula criteria^3^. Patients without postoperative records were considered lost to follow-up. Disease recurrence was confirmed through imaging when clinically suspected.

### Statistical analysis

Associations between diagnosis type, complications, recurrence, and death were assessed using the Chi-square test, likelihood-ratio test, and Fisher's exact test. Group means were compared using Student's t-test or ANOVA with Tukey's post-hoc test. For non-normally distributed variables, the Mann-Whitney U test or Kruskal-Wallis test, followed by Dunn's post-hoc test, was used. The Cox proportional hazards model was employed to identify prognostic factors related to DFS. Covariates with clinical relevance and P-values less than 0.100 were included in the multivariate analysis. Hazard ratios (HR) with 95% confidence intervals (CI) were calculated to evaluate associations. Postoperative mortality was analyzed separately from DFS to allow for a more accurate comparison of recurrence. Kaplan-Meier survival curves were generated, and the log-rank test was used to compare survival or DFS for clinically important variables. Statistical analyses were conducted using SPSS software (Version 19; SPSS Inc., Chicago, IL), with p-values less than 0.05 considered statistically significant.

## RESULTS

**Figure 1:**
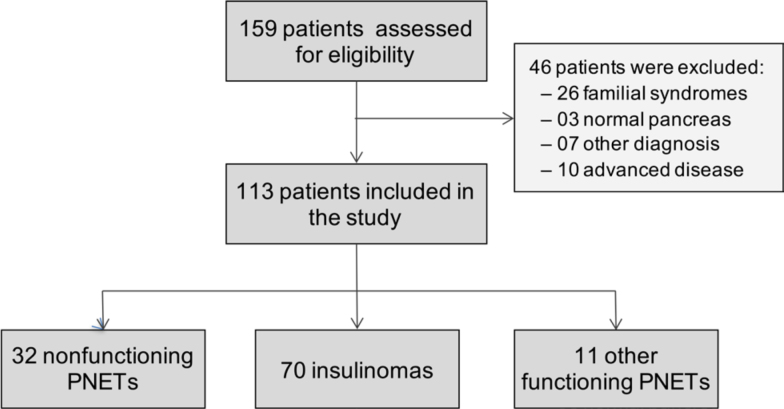
Flowchart of 159 patients initially assessed

Out of the 159 patients assessed, 113 were included in the study. Exclusion criteria are listed in Figure 1. Patients were classified as follows: NF-PNETs (28.3%), insulinomas (54.0%), and other functioning PNETS (OF-PNETs) (9.7%), including 4 glucagonomas, 2 gastrinomas, 1 gastrinoma-glucagonoma, 2 somatostatinomas, 1 adrenocorticotropin-producing tumor, and 1 carcinoid tumor diagnosed with diarrhea related to the pancreatic tumor.

PNTEs: Pancreatic neuroendocrine tumors.

### Clinical characteristics

Patients with insulinomas were younger, had a lower ASA score, fewer comorbidities per patient, and a higher BMI compared to those with NF-PNETs and OF-PNETs. There were no significant differences between the groups in terms of gender and KPS (Table 1).

**Table 1:** Clinical characteristics of patients with insulinoma, other functioning pancreatic neuroendocrine tumor, and nonfunctioning functioning pancreatic neuroendocrine tumor.

Characteristics		Insulinoma	OF-PNET	NF-PNET	p-value
		n=70	n=11	n=32	
Age: mean (SD), years		40.8 (16.5)	51.5 (17.1)	53.3 (17.9)	0.002
Gender: male, n (%)		33/70 (47.1)	6/11 (54.5)	13/32 (40.6)	0.693
BMI: mean (SD), Kg/m²		29.7 (6.3)*	25.4 (8.0)	26.6 (5.0)	0.042
KPS score: =90 *vs*. =80, n (%)		54/60 (90)	7/10 (70)	24/28 (85.7)	0.201
ASA score: I/II *vs*. III, n (%)		66/70 (94.3)*	8/11 (72.7)	26/32 (81.2)	0.029
Comorbidities: n (%)		34/68 (52.3)*	8/11 (72.7)	24/29 (82.8)	0.013
Types of surgery: n (%)					
	Enucleation	38/70 (54.3)*	1/11 (9.1)	4/32 (12.5)	<0.001
	Distal pancreatectomy	26/70 (37.1)	3/11 (27.3)	10/32 (31.3)	0.733
	Central pancreatectomy	4/70 (5.7)	1/11 (9.1)	5/32 (15.6)	0.263
	Pancreatoduodenectomy	0/70 (0)*	6/11 (54.5)	13/32 (40.6)	<0.001
	Subtotal pancreatectomy	2/70 (2.9)	0/11 (0)	0/32 (0)	0.535

OF-PNET: other functioning pancreatic neuroendocrine tumor; NF-PNET: nonfunctioning functioning pancreatic neuroendocrine tumor; SD: standard deviation; BMI: body mass index; KPS: Karnofsky performance score; ASA: American Society of Anesthesiologists; vs: versus.

* p<0.05: insulinoma ≠ from both other groups.

### Surgical techniques

Among the 113 patients, five types of pancreatic resections were performed: enucleation (38.1%), distal pancreatectomy (34.5%), central pancreatectomy (8.8%), pancreatoduodenectomy (PD) (16.8%), and subtotal pancreatectomy (1.8%). Patients with insulinomas underwent significantly more enucleations and fewer PDs compared to those with NF-PNETs and OF-PNETs. There were no significant differences between groups in the rates of distal and central pancreatectomies. Only two patients underwent subtotal pancreatectomy (Table 1).

Among the 39 patients who had a distal pancreatectomy, splenectomy was performed in 28 cases (82.4%). Splenic preservation was achieved in 61.5% of distal pancreatectomies for insulinomas, 47.4% for NF-PNETs, and 100% for OF-PNETs, with no statistically significant differences.

### Pathological findings

Most insulinomas were classified as grade I tumors (81.6%), whereas only 50.0% of NF-PNETs fell into this category. Insulinoma patients presented with significantly smaller tumor diameters and lower TNM stages compared to those with NF-PNETs and OF-PNETs. However, there was no significant difference in tumor grade between insulinomas and OF-PNETs (Table 2).

**Table 2:** Pathological characteristics according to pancreatic tumor type.

Characteristics	Insulinoma	OF-PNET	NF-PNET	p-value
	n=70	n=11	n=32	
WHO: grade 1 *vs.* other grades, n (%)	31/38 (81.6)*	4/8 (50)	15/30 (50)	0.009
Tumor size: median (IQR), cm	1.5 (1.1–2.0)†	4.5 (3–7)	3.2 (1.9–6.0)	<0.001
TNM AJCC: I *vs* ≥II, n (%)	65/68 (95.6)†	3/11 (27.3)	16/31 (51.6)	<0.001

OF-PNET: other functioning pancreatic neuroendocrine tumor; NF-PNET: nonfunctioning functioning pancreatic neuroendocrine tumor; WHO: World Health Organization 2022 grades, well differentiated PNET grade 1, 2, 3 and poorly neuroendocrine carcinoma (NEC)-small cell; *vs*: *versus*; IQR: interquartile range; TNM AJCC: Tumor, Node, Metastasis staging system from American Joint Committee on Cancer (9th Edition).

* p<0.05: insulinoma ≠ from NF-PNET;

† p<0.05: insulinoma ≠ from both other groups.

### Postoperative complications

The most common postoperative complication was POPF, which occurred in 27.1% of patients, with most POPFs (83.3%) classified as grade B. There were no significant differences between groups in the incidence of POPF, overall complications, or major complications (Table 3).

**Table 3:** Postoperative complications and follow-up of insulinoma, other functioning pancreatic neuroendocrine tumor, and nonfunctioning functioning pancreatic neuroendocrine tumor patients.

Characteristics	Insulinoma	OF-PNET	NF-PNET	p-value
	n=70	n=11	n=32	
Pancreatic fistula: n (%)	24/70 (34.3)	3/11 (27.3)	9/32 (28.1)	0.783
Any complication*: n (%)	48/70 (68.6)	7/11 (63.6)	17/32 (53.1)	0.309
Major complication (IIIb-V)*: n (%)	4/70 (5.7)	1/11 (9.1)	3/32 (9.4)	0.630
Length of hospital stay: median (IQR), days	13 (9–16.3)	11 (8–19)	10 (5-14.5)	0.082
Follow-up time: median (IQR), years	4.7 (1.8–9.8)	9.7 (5.1–16.3)	3.6 (0.1–7.2)‡	0.024
Recurrence: n (%)	4/65 (6.2)†	4/11 (36.4)	9/30 (30.0)	0.005
Time to recurrence: median (IQR), days	4.6 (1.5–6.6)	5.6 (1.7–11.3)	3.1 (2.7–4.3)	0.787
Death: n (%)	1/70 (1.4)	1/11 (9.1)	3/32 (9.4)	0.142

OF-PNET: other functioning pancreatic neuroendocrine tumor; NF-PNET: nonfunctioning functioning pancreatic neuroendocrine tumor; IQR: interquartile range.

* according to Clavien-Dindo classification;

† p <0.05: insulinoma ≠ from both other groups;

‡ p <0.05: NF-PNET ≠ from both other groups.

### Follow up and tumor recurrence

The median follow-up period was 4.9 years (IQR 1.41-8.5), with a significantly shorter follow-up time observed in the NF-PNET group compared to the Insulinoma and OF-PNET groups. Seven patients with less than 30 days of follow-up were excluded from the recurrence analysis. Of the 106 patients evaluated, 17 (16.0%) experienced tumor recurrence. The median time to recurrence following resection was 4.1 years (IQR 2.7-5.6), with no significant differences between groups. Recurrence was significantly lower after insulinoma resection (6.2%) compared to NF-PNET (30.0%) and OF-PNET (36.4%) resections. There were no significant differences in median hospital stay, follow-up time, or absolute number of deaths between groups (Table 3). No deaths were attributed to recurrence.

**Figure 2:**
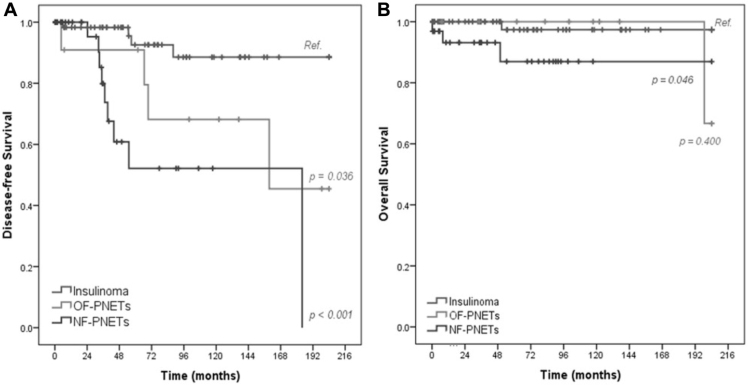
Survival Outcomes by Tumor Type. A) Disease-free survival stratified by tumor type: insulinoma, OF-PNET, and NF-PNET. B) Overall survival stratified by tumor type.

Patients with insulinomas showed greater DFS at 10 years (88.5%) compared to those with OF-PNETs (68.2%) and NF-PNETs (52.1%) (Figure 2A). Actuarial survival analysis indicated that patients with NF-PNETs had a 10-year survival of 86%, which was significantly lower than that of patients with insulinomas (97.4%) and OF-PNETs (100%) (Figure 2B). DFS was significantly higher for TNM stage IA compared to stages IIA and IIB, but with no difference from stage IIA.

PNTEs: Pancreatic neuroendocrine tumors.

### Univariate and multivariate analysis of risk factors for pancreatic neuroendocrine tumors recurrence

Univariate analysis identified age >60 years, tumor size, TNM stage II, and tumor types OF-PNET and NF-PNET as potential prognostic factors for disease recurrence. Multivariate analysis confirmed NF-PNET as an independent prognostic factor for recurrence (Table 4).

**Table 4:** Univariate and multivariate analysis for disease-free survival.

Disease-free survival		Univariate	95%CI	p-value	Multivariate†	95%CI	p-value
Characteristics		HR			HR		
Male *vs* female		1.33	0.50–3.53	0.570	-	-	-
Age >60 *vs*=60 years		2.73	1.05–7.10	0.040	1.49	0.51–4.40	0.466
ASA III *vs* ASA I/II		3.01	0.99–0.67	0.051	1.95	0.55–6.94	0.305
Comorbidities *vs* absent		1.96	0.63–6.08	0.246	-	-	-
Tumor size, cm		1.18	1.06–1.31	0.003	1.06	0.89–1.26	0.521
TNM≥II *vs* TNM I		3.67	1.42–9.54	0.007	1.26	0.29–5.40	0.757
POC* *vs* non-POC		0.95	0.33–2.72	0.925	-	-	-
Insulinoma *vs*							
	OF-PNET	4.07	1.01–16.36	0.048	2.04	0.36–11.49	0.417
	NF-PNET	8.38	2.55–27.55	< 0.001	5.43	1.38–21.36	0.016

HR: Hazard ratio; CI: confidence interval; ASA: American Society of Anesthesiology; TNM: tumor, node, and metastasis; POC: postoperative complications; OF-PNET=other functioning pancreatic neuroendocrine tumor; NF-PNET: nonfunctioning functioning pancreatic neuroendocrine tumor.

* according to Clavien-Dindo classification;

† variables with p<0.100 in the univariate analysis were included in the multivariate model.

## DISCUSSION

This study classified PNETs treated with curative intent into three categories: nonfunctioning PNETs (NF-PNETs), insulinomas, and OF-PNETs. This classification aimed to facilitate results analysis, considering the high biological variability among tumor types. Histological type significantly impacts prognosis; for instance, up to 90% of insulinomas are benign, whereas NF-PNETs and OF-PNETs exhibit higher incidence of malignancy^16,30^. Even small sporadic NF-PNETs=2 cm can present lymph node or distant metastases in 8, to 14% of cases, and for tumors >2 cm, metastases incidence increases to 34 to 53%^9,15^.

In this cohort, insulinomas comprised 62% of cases, a proportion higher than NF-PNETs, contrasting with prior studies reporting a higher incidence of NF-PNETs^16,26^. This may be attributed to frequent referrals of insulinomas patients from the Endocrinology Service to the Pancreato-Biliary Surgery Service. Over the past decade, the proportion of operated NF-PNETs has risen, likely due to improved imaging techniques that detect asymptomatic tumors more frequently. This trend aligns with other studies, where 46% of resected PNETs were nonfunctioning tumors^38^. Insulinomas are the most common functional PNETs, accounting for 80–90% of cases^20,35^, and in this study, they represented 86% of functioning tumors.

The incidence of PNETs generally peaks between the fourth and sixth decades of life^16,20,30^, with insulinoma patients presenting at younger ages^4,13^. In the present study, insulinoma patients had a mean age of 41 years, compared to 52 and 53 years for OF-PNET and NF-PNETs patients, respectively. Additionally, insulinoma patients exhibited higher BMI, possibly due to hypoglycemia-induced neuroglycopenic episodes that lead to increased caloric intake. Younger age and lower comorbidity rates likely contributed to higher performance status and lower ASA scores within this group.

Surgery remains essential for PNET cure, with treatment decisions influenced by tumor location, size, and nature. For nonfunctioning sporadic PNETs, surveillance is debated for tumors 1–2 cm in size, with some opting for a conservative approach in selected cases. Tumors ≤3 cm can be enucleated in selected cases without suspicion of lymph node and distant metastasis, while larger NF-PNETs typically require resection with regional lymphadenectomy due to the higher risk of lymph node metastasis^23–25^. In this study, 47% of patients underwent pancreatic parenchyma-sparing techniques, such as enucleations and central pancreatectomies. Among these cases, three were NF-PNETs larger than 3 cm, with one patient experiencing recurrence after 15 years of follow-up. As expected, the majority of patients (79%) who underwent parenchyma-sparing procedures were diagnosed with insulinomas, given the typical benign nature and lower metastatic risk of these tumors.

Within this insulinoma group, 54% of cases were managed with enucleation and 37% underwent distal pancreatectomies. PD was not performed on any insulinoma patients, as this extensive procedure is rarely indicated for insulinomas^19^. Enucleation is generally preferred for insulinomas; however, this technique is contraindicated for tumors located within 3 mm of the main pancreatic duct due to the risk of ductal injury or when malignancy is suspected^19^. Over recent decades, minimally invasive pancreatic surgery has gained popularity, with PNETs among the primary indications for this approach. Minimally invasive techniques, especially distal pancreatectomy, have shown favorable outcomes for PNETs, particularly when tumor location and size are conducive to a less invasive procedure^7,21,42^.

Although distal pancreatectomy with splenic preservation is a safe procedure, it is generally recommended for benign tumors. Splenic preservation helps avoid the risk of post-splenectomy sepsis; however, it may compromise adequate lymphadenectomy for malignant PNETs, limiting the thoroughness of oncologic resection^8^. In this study, 28% of distal pancreatectomies were performed with splenic preservation, predominantly for insulinomas. Other functioning PNETs, due to their higher malignant potential, generally underwent more extensive resections, except for one 2 cm gastrinoma, which was enucleated, consistent with PNETs guidelines^19,24^. Nevertheless, functioning PNETs with a higher likelihood of malignancy are preferably managed with resection combined with regional lymphadenectomy^30^.

Pancreatic surgery currently has a low mortality rate; however, the overall incidence of postoperative complications remains highly variable, ranging from 30 to 60%^27^. This variability suggests a lack of standardization with underreporting of minor complications. Due to the absence of a consensus on complication reporting, the Clavien-Dindo classification was adopted in this study^10^. Here, 64% of patients experienced postoperative complications, but only 7% were classified as major complications (grade IIIb-V). POPF, often considered the "Achilles heel" of pancreatic surgery, was the most common complication, with an incidence of 32% in this study — slightly higher than the reported range of 5 to 26%^28^. Nonetheless, most of these cases were POPF classified as grade B, indicating a less severe form. Additionally, biliary fistula was observed in 2.7% of cases, primarily associated with PDs, although one instance occurred following enucleation. This is consistent with the literature, where biliary fistula after PD is reported at around 3 to 8%^2^.

The overall death rate in this study was 4.4% and was not associated with tumor recurrence, with a median follow-up of 4.9 years. However, late mortality may be underestimated, as 21% of patients were lost to follow-up within the first year. Patients with functioning tumors showed significantly higher 10-year survival rates compared to those with nonfunctioning tumors. Additionally, patients with nonfunctioning tumors had a significantly shorter follow-up period than those with functioning tumors.

Two factors likely contributed to these differences in the follow-up duration: variations in survival rates between the groups and the recent increase in diagnoses of resectable nonfunctioning tumors, driven by advances in imaging techniques, while the diagnosis rate for functioning tumors has remained stable^26^. Importantly, mortality was not related to tumor recurrence, as all 17 patients with tumor recurrence remained alive throughout the study period.

PNETs display a broad spectrum of malignancy, with 5-year survival rates between 44 and 87%^14,33^. Insulinomas are typically of lower grade, diameter, and TNM staging, with only 5–10% showing malignancy^1,11,36^. In this study, 66% of resected PNETs were graded 1 and 76% were staged as TNM IA/IB, suggesting a lower risk of recurrence. This hypothesis is supported by the finding of significantly higher DFS for TNM stage IA/IB compared to other stages. The WHO classification designates grade 1 tumors as well-differentiated and generally associated with a better prognosis. However, some decrease in actuarial survival has been observed over time, suggesting that these tumors cannot be assumed to have a complete benign behavior from the outset^12,15^. Ye et al.^40^, in a series of 138 patients, observed that WHO staging does not accurately distinguish the prognosis of patients with regional and distant metastasis in neuroendocrine tumors.

In this study, the overall incidence of recurrence was 16%. Nonfunctioning tumors had the worst prognosis, with a 10-year DFS of 52.1%. Conversely, patients with insulinomas have the best prognosis, with a 10-year DFS of 86.5%. Substantial evidence suggests a poorer prognosis for nonfunctioning PNETs; for example, a study involving 2,158 patients reported a 10-year survival of only 17% for nonfunctioning tumors^16^.

In this investigation, prognosis was evaluated in relation to disease recurrence. In the univariate analysis, age >60 years, tumor size, TNM ≥II, and NF-PNET diagnosis emerged as potential prognostic indicators for disease recurrence. However, in the multivariate analysis, only the diagnosis of NF-PNET remained an independent risk factor for recurrence. A SEER database study suggests that tumor functional state is a prognostic indicator^17^, while other studies highlight tumor grade, tumor size, and metastasis as significant prognostic factors^12,39^. Interestingly, a more recent SEER database study indicated that functioning tumors may be associated with a worse prognosis. This study, however, demonstrated a strong correlation between NF-PNET and poorer prognosis in multivariate analysis, as evidenced by a lower 10-year DFS.

## CONCLUSIONS

This study highlights the diverse prognostic outcomes among PNETs based on tumor functionality and staging. Although complete surgical resection offers significant survival benefits, NF-PNETs demonstrate a higher risk of recurrence, even after curative intent surgery. The findings emphasize that NF-PNETs are independently associated with a poorer prognosis, as shown by lower 10-year DFS. While age, tumor size, and TNM stage II emerged as prognostic factors in univariate analysis, only NF-PNET status remained significant in multivariate analysis.

These results underscore the importance of long-term follow-up, especially for NF-PNETs patients, to enable early recurrence detection and optimize management strategies. Further research is necessary to refine risk stratification and treatment protocols tailored to the unique characteristics of PNET subtypes.

Central Message Pancreatic neuroendocrine tumors (PNETs) exhibit diverse behaviors and varying risks of recurrence depending on tumor type and stage. This study reveals that non-functioning PNETs (NF-PNETs) have a significantly higher recurrence rate post-surgery, challenging the assumption of their indolent nature. These findings underscore the importance of developing tailored, risk-specific follow-up strategies. Enhanced surveillance for NF-PNET patients could facilitate early recurrence detection, enable timely interventions, and potentially improve long-term outcomes. This research highlights the need for a personalized approach in postoperative care for PNET patients, aiming to optimize management and quality of life.

Perspectives This study highlights the importance of personalized follow-up for non-functioning pancreatic neuroendocrine tumors (NF-PNETs) due to their elevated recurrence risk. A future direction could involve identifying and validating biomarkers to refine prognosis and personalize care. Biomarkers like DAXX, ATRX, and Ki-67 provide insight into tumor aggressiveness and differentiation. Additionally, markers such as PTEN, TSC2, CK19, KIT, p53, and Rb could help differentiate high-grade, well-differentiated NETs from neuroendocrine carcinomas. These molecular distinctions allow for more precise prognostic stratification and could guide individualized treatment approaches, ultimately enhancing long-term outcomes for patients.
